# Erythrocyte–brain endothelial interactions induce microglial responses and cerebral microhemorrhages in vivo

**DOI:** 10.1186/s12974-023-02932-5

**Published:** 2023-11-15

**Authors:** Hai Zhang, Rachita K. Sumbria, Rudy Chang, Jiahong Sun, David H. Cribbs, Todd C. Holmes, Mark J. Fisher, Xiangmin Xu

**Affiliations:** 1 Department of Anatomy and Neurobiology, School of Medicine, University of California, Irvine, CA 92697 USA; 2 Department of Biomedical and Pharmaceutical Sciences, School of Pharmacy, Chapman University, Irvine, CA 92618 USA; 3 Department of Neurology, University of California, Irvine, CA 92697 USA; 4 Institute for Memory Impairments and Neurological Disorders, University of California, Irvine, CA 92697 USA; 5 Department of Physiology and Biophysics, School of Medicine, University of California, Irvine, CA 92697 USA; 6 Center for Neural Circuit Mapping, University of California, Irvine, CA 92697 USA; 7 Beckman Laser Institute, University of California, Irvine, CA 92697 USA; 8 Department of Pathology & Laboratory Medicine, University of California, Irvine, CA 92697 USA; 9 Department of Biomedical Engineering, University of California, Irvine, CA 92697 USA

## Abstract

**Background:**

Cerebral microhemorrhages (CMH) are associated with stroke, cognitive decline, and normal aging. Our previous study shows that the interaction between oxidatively stressed red blood cells (RBC) and cerebral endothelium may underlie CMH development. However, the real-time examination of altered RBC–brain endothelial interactions in vivo, and their relationship with clearance of stalled RBC, microglial responses, and CMH development, has not been reported.

**Methods:**

RBC were oxidatively stressed using tert-butylhydroperoxide (t-BHP), fluorescently labeled and injected into adult Tie2-GFP mice. In vivo two-photon imaging and ex vivo confocal microscopy were used to evaluate the temporal profile of RBC–brain endothelial interactions associated with oxidatively stressed RBC. Their relationship with microglial activation and CMH was examined with post-mortem histology.

**Results:**

Oxidatively stressed RBC stall significantly and rapidly in cerebral vessels in mice, accompanied by decreased blood flow velocity which recovers at 5 days. Post-mortem histology confirms significantly greater RBC–cerebral endothelial interactions and microglial activation at 24 h after t-BHP-treated RBC injection, which persist at 7 days. Furthermore, significant CMH develop in the absence of blood–brain barrier leakage after t-BHP-RBC injection.

**Conclusions:**

Our in vivo and ex vivo findings show the stalling and clearance of oxidatively stressed RBC in cerebral capillaries, highlighting the significance of microglial responses and altered RBC–brain endothelial interactions in CMH development. Our study provides novel mechanistic insight into CMH associated with pathological conditions with increased RBC–brain endothelial interactions.

**Supplementary Information:**

The online version contains supplementary material available at 10.1186/s12974-023-02932-5.

## Introduction

Cerebral microhemorrhages (CMH), the pathological substrates of cerebral microbleeds (CMB), are microscopic accumulations of blood degradation products in the brain. CMH can be identified by magnetic resonance imaging (MRI) detection of ferromagnetic iron, and histologically by using Prussian blue staining that detects hemosiderin-iron. CMH are common findings in hypertension, cerebral amyloid angiopathy, Alzheimer’s disease, chronic kidney disease, cerebrovascular disease, and normal aging [1–4]. Age is the most significant independent risk factor for CMB, with increasing CMB prevalence among older populations [5, 6]. CMB are associated with increased risk of ischemic and hemorrhagic stroke, cognitive decline, and dementia [7–9], making them increasingly clinically relevant for aging populations.

Despite considerable interest in CMH, the exact mechanisms underlying their formation are not fully elucidated [10]. CMH occur at the cerebral capillary and arteriolar levels [3, 10] and are understood to arise from brain vessel wall disruption leading to extravasation of red blood cells (RBC) into brain parenchyma [2, 11]. Once within brain parenchyma, RBC can be phagocytosed by microglia, which orchestrates the release of hemoglobin and subsequent heme and iron from RBC [12]. Iron derived from extravasated RBC then produce CMB signatures, which are identified using MRI or post-mortem histology [13]. In addition, disruption in brain iron homeostasis and the release of iron following ischemic damage can produce CMH [11, 13]. Our recent work shows the involvement of brain endothelial erythrophagocytosis in producing CMH [14, 15].

While brain endothelial cells are not categorized as professional phagocytes, their phagocytic phenotype has been reported in vitro and in rodent models [16–18]. Specifically, the erythrophagocytic phenotype of brain endothelium makes this phenomenon relevant to CMH development. Our previous work using in vitro and in vivo rodent models shows that oxidatively stressed RBC can adhered to and be engulfed by brain endothelium [14, 15, 19]. This increased brain endothelial erythrophagocytosis is associated with increased abluminal presence of iron-rich hemoglobin and iron across an intact endothelial monolayer in vitro, and with CMH signatures in vivo in the absence of brain endothelial dysfunction [14, 15]. However, the real-time examination of altered RBC–brain endothelial interactions in vivo, and its relationship with clearance of stalled RBC, microglial responses, and CMH development, has not been reported.

In the current study, RBC were artificially aged using tert-butylhydroperoxide (t-BHP), an oxidative stressor known to induce changes consistent with RBC aging and rheological changes observed under pathological conditions [16, 20–22]. Fluorescently labeled t-BHP- or phosphate-buffered saline (PBS)-treated RBC were injected into adult Tie2-GFP mice that express green fluorescent protein (GFP)-labeled endothelial cells. In vivo two-photon imaging was used to evaluate the temporal profile of RBC–brain endothelial interactions and changes in cerebral blood flow velocity associated with altered RBC–brain endothelial interactions. Two-photon microscopy analysis was followed up with post-mortem histological analysis of brain sections for a comprehensive examination of altered RBC–brain endothelial interactions, extravasation of t-BHP-treated RBC into the brain parenchyma, microglial response, and CMH development. Our results demonstrate that t-BHP-treated RBC stall in brain blood vessels rapidly after being injected in mice. Mice injected with t-BHP-treated RBC show significantly increased RBC–brain endothelial interactions at the cerebral capillary level that coincide with persistent microglial responses and significant CMH burden, without overt blood–brain barrier (BBB) leakage. Our in vivo and ex vivo findings show the stalling and clearance of oxidatively stressed RBC in cerebral capillaries, highlighting the significance of microglial responses and altered RBC–brain endothelial interactions in CMH development.

## Materials and methods

### Animals

Male Tie2-GFP mice and genetic background matched FVB/NJ mice were purchased from the Jackson Laboratory (Bar Harbor, ME, USA). Mice were acclimated for one week in the vivarium before experiments and had free access to food and water. The temperature of the vivarium was maintained between 21 and 23 °C, and humidity was maintained between 40% and 70%. All the animal experiments were approved by the University of California, Irvine, Institutional Animal Care and Use Committee and carried out in accordance with the Guide for the Care and Use of Laboratory Animals.

### Erythrocyte purification and treatment

Blood was harvested from anesthetized 8- to 12-week-old male FVB/NJ mice into heparinized tubes and centrifuged at 500 × g for 10 min at 4 °C. The RBC-rich pellet was washed and resuspended three times with sterile phosphate-buffered saline (PBS without Ca^2+^ and Mg^2^^+^; Invitrogen, Waltham, MA, USA) while discarding the supernatant. RBC were counted and 1 × 10^9^ RBC were treated with either PBS or 3 mM t-BHP (Sigma-Aldrich, St. Louis, MO, USA) for 30 min in a water bath at 37 °C, spun down at 500 × g for 10 min, and counted using a hemocytometer to derive the desired cell concentration for labeling [14, 15]. t-BHP treatment was used to stimulate phosphatidylserine externalization and reduce RBC deformability and membrane CD47 to artificially age RBC in vitro [14–16, 22, 23].

### PKH26 labeling and RBC injection

Treated RBC were labeled with PKH26 Red Fluorescent Cell Linker Kit (Sigma, St. Louis, MO, USA) as per vendor instructions. PKH26 was selected due to its long elution half-life from labeled RBC which is optimal for in vivo studies [24]. Briefly, RBC were washed with sterile Dulbecco’s modified Eagle’s medium (DMEM; American Type Culture Collection, Manassas, VA, USA) (without fetal bovine serum (FBS)) and spun down at 400 × g for 5 min prior to discarding the supernatant. PKH26 stain was added to the RBC (at 4 µL of stain for every 2.5 × 10^8^ RBC in a total of 2 mL of diluent with cells) and stained in the dark for 4 min, before inactivating with an equal volume (2 mL) of DMEM with 10% FBS to the stain volume for one minute. After spinning at 400 × g for 10 min, the supernatant was discarded, RBC were resuspended in fresh sterile DMEM with 10% FBS and then counted using a hemocytometer. A total of 5 × 10^8^ to 1 × 10^9^ of PKH26-labeled RBC suspended in 150 µL of sterile saline were injected intravenously (retro-orbital) into male Tie2-GFP mice (10–12-week-old).

### Craniotomy

To perform in vivo imaging, we made a round cranial window over the somatosensory (S1) area. Tie2-GFP mice were anesthetized with isoflurane and placed on a stereotaxic frame. A midline incision was made in the scalp and the soft tissue on the skull was cleaned with a cotton tip. After locating the bregma, a 4-mm-diameter circle was drawn on the skull of the right side, centered at anterior posterior (AP) − 0.5 mm and lateral (L) 2.5 mm. Then the cranial window was carefully drilled to avoid damaging the dura. Gel foam was applied to stop bleeding when necessary. A piece of round cover glass was placed on the window and secured in place with super glue and dental cement. A head bar was attached to the skull with dental cement. Mice were subcutaneously injected with 3 mg/kg carprofen as analgesia for 3 days after surgery.

### Two-photon imaging and data processing

To visualize time-lapsed events that occur between GFP-positive brain endothelium and PBS- or t-BHP-treated PKH26-labeled RBC, RBC and cerebral blood vessels were longitudinally imaged at designated time points (1–4 h, 24 h, 5 days, and 7 days post-injection). The same area or same blood vessels were captured when possible by image co-registration. Mice were anesthetized with isoflurane and placed under a two-photon microscope (Neurolabware, Los Angeles, CA, USA). Imaging was performed with 920 nm laser (Coherent, Palo Alto, CA, USA), images of blood vessels were collected after a 510/84 nm filter, and images of RBC were collected after a 607/70 nm filter. Two regions of 600 × 400 µm^2^ size, z-depth of 120–180 µm were scanned at 2 µm step with a 16 × objective for each mouse. The frame rate was 16 frames/s. For each z step, 160 frames were acquired and averaged, then maximum z-projection was created. For the RBC imaging, final images were processed to keep stalled RBC only and flowing RBC were removed. In each imaged area, a few capillaries were recorded at high resolution to image single RBC flow for 3–5 min, and the frame rate of the high-resolution imaging was 32 frames/s. Number of total blood vessel branches and number of branches with RBC stalls were counted to calculate the percentage of RBC-stalled vessels. To quantify the blood flow velocity, the movement of RBC across consecutive frames was analyzed and the speed of flow was calculated from distance/time. Approximately 100 branches were analyzed for each mouse.

### Brain harvest, cryopreservation, and sectioning

Mice were deeply anesthetized with isoflurane. Cardiac perfusions were performed utilizing ice-cold PBS for 5 min to clear the cerebral vasculature. For the 24-h study, the left cerebral hemisphere was snap frozen, while the right cerebral hemisphere was fixed with 4% paraformaldehyde (PFA) in PBS for 24 h, followed by a 24-h serial incubation in 10%, 20%, and 30% sucrose solution at 4 °C, frozen, mounted in Tissue-Tek OCT compound (Fisher Scientific, Hampton, NH, USA) and sliced into 20-µm-thick sagittal sections at − 22 °C using a Leica CM1850 cryostat (Micron Instruments, San Marcos, CA, USA). Three sections per mouse, approximately 600 μm apart, were used for direct imaging (post-sectioning without immunostaining) of the GFP-positive brain endothelium and PKH26-labeled RBC, and immunostaining. For the 7-day study, whole brains were fixed in 4% PFA and placed in a series of sucrose solutions for cryopreservation, mounted as described above, and then sliced into 20-µm-thick coronal sections at − 22 °C using the cryostat. Four sections per mouse, approximately 600 µm apart, were used for direct imaging (post-sectioning without immunostaining) of the GFP-positive brain endothelium and PKH26-labeled RBC, and immunostaining of Iba1 or fibrinogen.

### Confocal imaging and quantification

To visualize the GFP-positive brain endothelium and PKH26-labeled RBC postmortem, brain sections were mounted onto glass slides, cover-slipped using VectaMount aqueous mounting media (Vector Laboratories, Newark, CA, USA) and imaged using a Nikon ECLIPSE Ti-E confocal microscope (Nikon Instruments Inc., Melville, NY, USA) interfaced to the NIS-Elements software (Nikon Instruments), with a blue laser to visualize the GFP-positive brain endothelium and green laser to visualize the PKH26-labeled RBC. Three to four images per brain section from cortical and subcortical regions were acquired at a 40 × magnification under oil immersion on the confocal microscope to quantify the total number of cerebral vessels, diameter of vessels with RBC stalls, number of cerebral vessels positive for RBC stalls expressed as a percentage of total number of cerebral vessels, and extravasated RBC, per field of view using the NIH Image J software 1.50-jv6 (NIH, Bethesda, MD, USA) by an observer blinded to the experimental groups.

### Iba1 immunostaining and quantification

For Iba1 (ionized calcium-binding adapter molecule1) immunostaining (to detect microglia), free-floating brain sections were washed with PBS, incubated for 60 min with PBS containing 0.5% bovine serum albumin (BSA) and 0.3% Triton-X100 to block non-specific protein binding, and then incubated overnight at 4 °C with a rabbit anti-Iba1 antibody (1:500 dilution, Wako Chemicals USA, Richmond, VA, USA). The next day, sections were washed in PBS and then incubated for 2 h in the dark at room temperature with a donkey anti-rabbit IgG H&L with Alexa Fluor^®^ 647 secondary antibody (1:1000 dilution, Thermo Fisher Scientific, Waltham, MA, USA). Sections were then washed in PBS and distilled water before mounting and cover-slipping on glass slides. Four images per brain section were acquired at a 40 × magnification to quantify the total Iba1-positive immunoreactive area, total number of microglia, number of resting (ramified-like) and activated (amoeboid-like) microglia, and the number of cerebral blood vessels positive for RBC stalls and microglia, using NIH Image J software 1.50-jv6 by an observer blinded to the experimental groups.

### Prussian blue/iron staining and quantification

Prussian blue staining was used to visualize differences in the iron deposited in the brains of mice injected with PBS- or t-BHP-treated RBC [25]. For this, brain sections were mounted on glass slides, dried, washed with PBS, stained for 3 min with an iron staining solution (mixture of equal volumes of potassium ferrocyanide solution and hydrochloric acid solution; Abcam, Cambridge, UK). After removing the staining solution, the slide was gently washed in distilled water, counterstained for 5 min with nuclear fast red solution (Abcam, Cambridge, UK), rinsed in distilled water, dehydrated with alcohol, cleared, and sealed. Slides were imaged using a light microscopy at a 40 × magnification and the Prussian blue-positive area was quantified using the NIH Image J software 1.50-jv6 by an observer blinded to the experimental groups.

### Fibrinogen staining and quantification

To visualize BBB disruption, fibrinogen immunostaining was performed. Brain sections were washed with PBS, incubated for 60 min with PBS containing 0.5% BSA and 0.3% Triton-X100 to block non-specific protein binding, and then incubated overnight at 4 °C with a sheep anti-fibrinogen antibody (1:300, USBio, Metairie, LA, USA). The next day, sections were washed in PBS and then incubated for 2 h in the dark at room temperature with a donkey-anti-sheep IgG Alexa Fluor 647 (1:500, Thermo Fisher Scientific, Waltham, MA, USA) secondary antibody. Sections were then washed in PBS and distilled water before mounting and cover-slipping on glass slides. Four images per brain section were acquired at a 40 × magnification using a Nikon ECLIPSE Ti-E confocal microscope (Nikon Instruments) to quantify the total fibrinogen-positive immunoreactive area (%) per image (this includes vascular and extravascular fibrinogen) and extravasated fibrinogen-positive sites (a fibrinogen-positive site was defined as a collection of fibrinogen immunoreactive areas within a 20-µm-diameter circle in brain parenchyma; this parameter measures extravascular fibrinogen only) using the NIH Image J software 1.50-jv6 by an observer blinded to the experimental groups. Since fibrinogen staining detects both fibrin and fibrinogen, intravascular staining in the current study was used as an indicator of possible intravascular thrombus formation due to RBC aggregation.

### Statistical analysis

We used the linear mixed effect (LME) model to analyze the multiple measures from the same animal. Accordingly, data from multiple in vivo or brain section images per mouse were clustered per experimental group [26, 27], and analyzed using treatment as a fixed effect and mouse as a random effect. Data are presented as mean ± SEM of individual images per mouse per experimental group and statistical analysis was conducted in the Matlab software. *p* < 0.05 was considered statistically significant.

## Results

### t-BHP-treated RBC stall significantly in cerebral blood vessels, which significantly reduces blood flow velocity in vivo

**Fig. 1:**
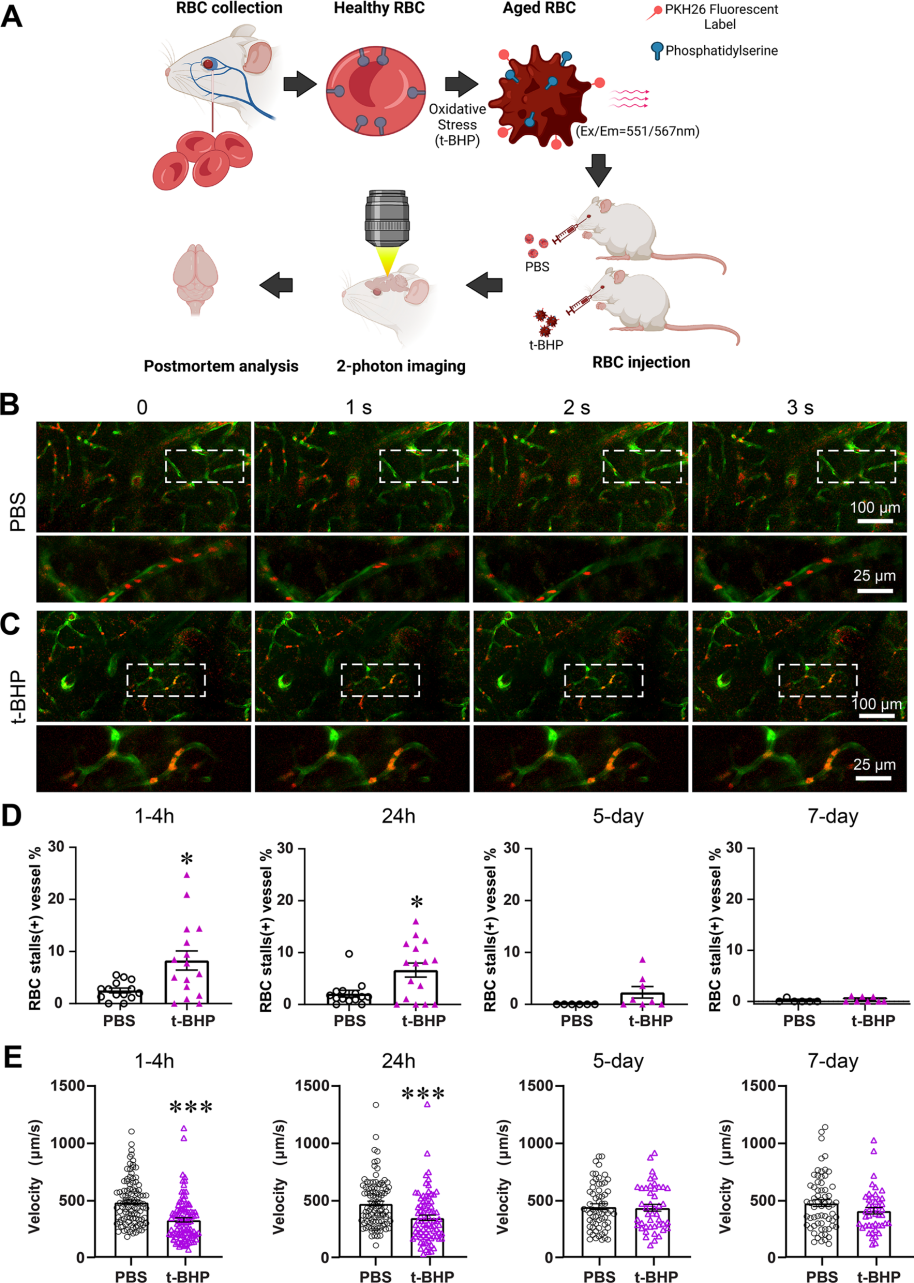
t-BHP-treated RBC stall in cerebral blood vessels and impair cerebral blood flow velocity shown by in vivo high-resolution two-photon imaging in mice. Schematic of the experimental design (**A**). RBC were collected from FVB/NJ mice, treated with control PBS or the oxidative stressor t-BHP, then labeled with the PKH26 fluorescent dye and injected intravenously into mice with Tie2-GFP-labeled endothelial vasculature. RBC (red) and cerebral blood vessels (green) were imaged in vivo using two-photon microscopy. Representative frames are shown at 1-s interval durations from control PBS-treated RBC-injected mice (**B**) and t-BHP-treated RBC-injected mice (**C**). The boxed areas are shown in the bottom panels of B and C, imaged at higher resolution and at different second intervals from parent images. RBC from the control PBS group exhibit robust flow in the cerebral blood vessels. In contrast, t-BHP-treated RBC stall significantly in cerebral blood vessels. Percentage of cerebral blood vessels with stalled RBC (**D**) and blood flow velocity (**E**) were measured at 1–4 h, 24 h, 5 days and 7 days after control versus t-BHP-treated RBC injections. Significantly more RBC stalls and reduced cerebral blood flow velocity are measured in mice injected with t-BHP-treated RBC relative to control PBS-treated RBC. Data are expressed at mean ± SEM and statistically analyzed with the LME. *p < 0.05, ***p < 0.001

Control PBS- or t-BHP-treated RBC were injected into Tie2-GFP mice, and in vivo imaging was performed at 1–4 h, 24 h, 5 days and 7 days post-injection to evaluate interactions between PKH26-labeled RBC and cerebral blood vessels labeled by Tie2-GFP using two-photon intra-vital microscopy (Fig. 1A). In vivo two-photon imaging shows no RBC stalls and robust blood flow in the cerebral blood vessels of mice injected with PBS-treated RBC (Fig. 1B, Additional file 2: video 1, Additional file 3: video 2). However, stalled RBC in cerebral blood vessels, including capillaries and larger vessels, are seen in mice injected with t-BHP-treated RBC, and such stalled RBC are observed at significantly greater levels relative to those seen in mice injected with control PBS-treated RBC at 1–4 h and 24 h after injection. Stalled RBC occur mostly in the capillaries of the brain (Fig. 1C, Additional file 4: video 3, Additional file 6: video 5, Additional file 8: video 7), but are also sometimes observed in relatively larger blood vessels (Additional file 1: Figure S1A, Additional file 10: video 9). Quantification of the number of cerebral blood vessels positive for RBC stalls indicates that mice injected with t-BHP-treated RBC have significantly more cerebral blood vessels with RBC stalls relative to controls between 1 and 24 h after RBC injection (Fig. 1D, 1–4 h: 8.3 ± 1.8% vs. 2.5 ± 0.5% in PBS group, *p* < 0.05; 24 h: 6.6 ± 1.4% vs. 2.1 ± 0.7% in PBS group, *p* < 0.05). At 5 days and 7 days after RBC injection, we observe very few RBC stalls relative to the numbers we see at 24 h after t-BHP-treated RBC injection. There are no differences in the percentage of cerebral blood vessels with stalled RBC comparing control PBS- and t-BHP-treated RBC-injected mice measured by in vivo imaging experiments at 5 or 7 days (Fig. 1D, 5 days: 2.3 ± 1.1% vs. 0 in PBS group; 7 days: 0.13 ± 0.13% vs. 0.37 ± 0.18% in PBS group) (Additional file 1: Figure S1B, C). We also measured the velocity of cerebral capillary blood flow in vivo using two-photon imaging (Fig. 1E). At 1–4 h and 24 h after RBC injection, the blood flow velocity in t-BHP-treated RBC-injected mice is significantly lower compared to control PBS-treated RBC-injected mice (1–4 h: 323.0 ± 18.8 vs. 479.7 ± 19.4 µm/s in PBS group, *p* < 0.001; 24 h: 344.7 ± 24.0 vs. 469.5 ± 20.8 µm/s in PBS group, *p* < 0.001). At 5 days and 7 days after RBC injection, the cerebral blood flow velocity is comparable between PBS- and t-BHP-RBC-injected mice (5 days: 429.0 ± 29.8 vs. 438.7 ± 25.1 µm/s in PBS group; 7 days: 397.0 ± 28.3 vs. 464.3 ± 29.7 µm/s in PBS group) without significant differences. These results suggest clearance mechanisms that facilitate recovery of cerebral blood flow velocity.

**Fig. 2:**
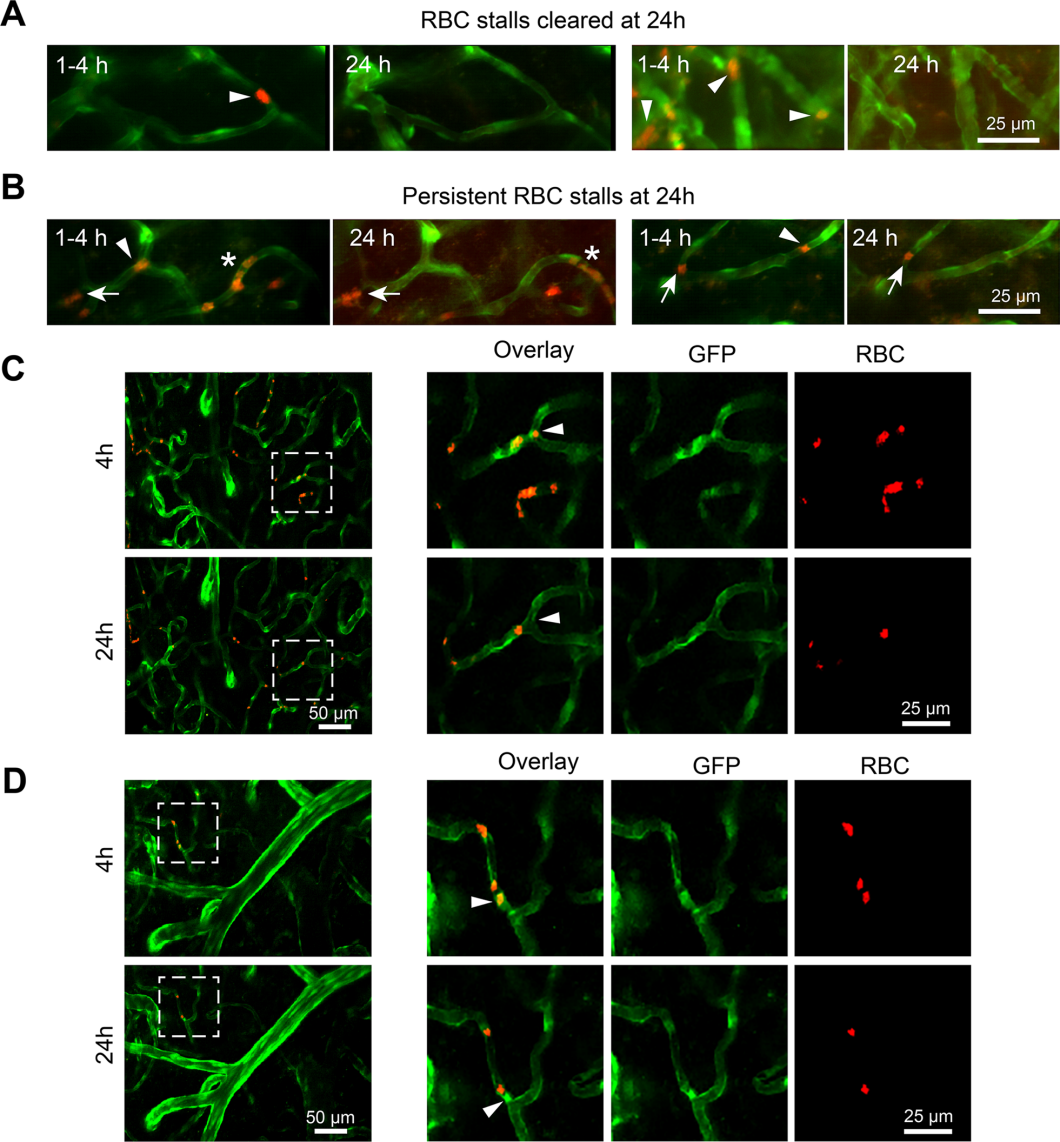
The majority of t-BHP-treated RBC stalls are cleared by 24 h after injection. RBC stalls are dynamic (**A, B**). Repeated in vivo imaging of the same capillaries at 1–4 h and 24 h after t-BHP-RBC injections shows that most stalled RBC are cleared by 24 h after injection (**A**, **B** arrowheads). A few cerebral vessels show persistent RBC stalls at the same location at 1–4 h and 24 h (**B**, arrows), or at different locations (**B**, asterisk). Examples of t-BHP-treated RBC stalled in cerebral blood vessels at 4 h and the eventual clearance of those stalled RBC 24 h after injection (**C, D**). The boxed areas are shown as higher resolution images in the right panels of C and D. Images show that RBC adhere to brain endothelial cells (arrowheads, red RBC overlapping with the green endothelial cells) at 4 h after injection (top rows). At 24 h at the same cerebral blood vessel site (bottom rows), the stalled RBC have cleared. Note in **D**, the arrowhead indicates that RBC appear to move toward the branch from 4 to 24 h imaging

We performed in vivo imaging of the same field longitudinally and find that RBC stalls following t-BHP-treated RBC injection are dynamic over time. Most RBC stalls are cleared by 24 h post-injection (Fig. 2A, Additional file 6: video 5, Additional file 7: video 6), but a few vessels show persistent RBC stalls at the same or different locations (Fig. 2B, Additional file 4: video 3, Additional file 5: video 4, Additional file 8: video 7, Additional file 9: video 8). Furthermore, by careful examination of interactions between brain endothelium and RBC, we find that some stalled t-BHP-treated RBC are co-localized with brain endothelial cells at 4 h after injection and those co-localized RBC are cleared by 24 h post-injection (Fig. 2C, D, Additional file 1: Figure S2). In vivo imaging therefore reveals that t-BHP-treated RBC stall in cerebral vessels rapidly after injection and induce decreased cerebral blood flow velocity, an effect that appears to be mitigated eventually by clearance of RBC stalls from the circulation.

### Post-mortem assessment confirms robust brain endothelial-RBC interactions within 24 h after t-BHP-treated RBC injection

**Fig. 3:**
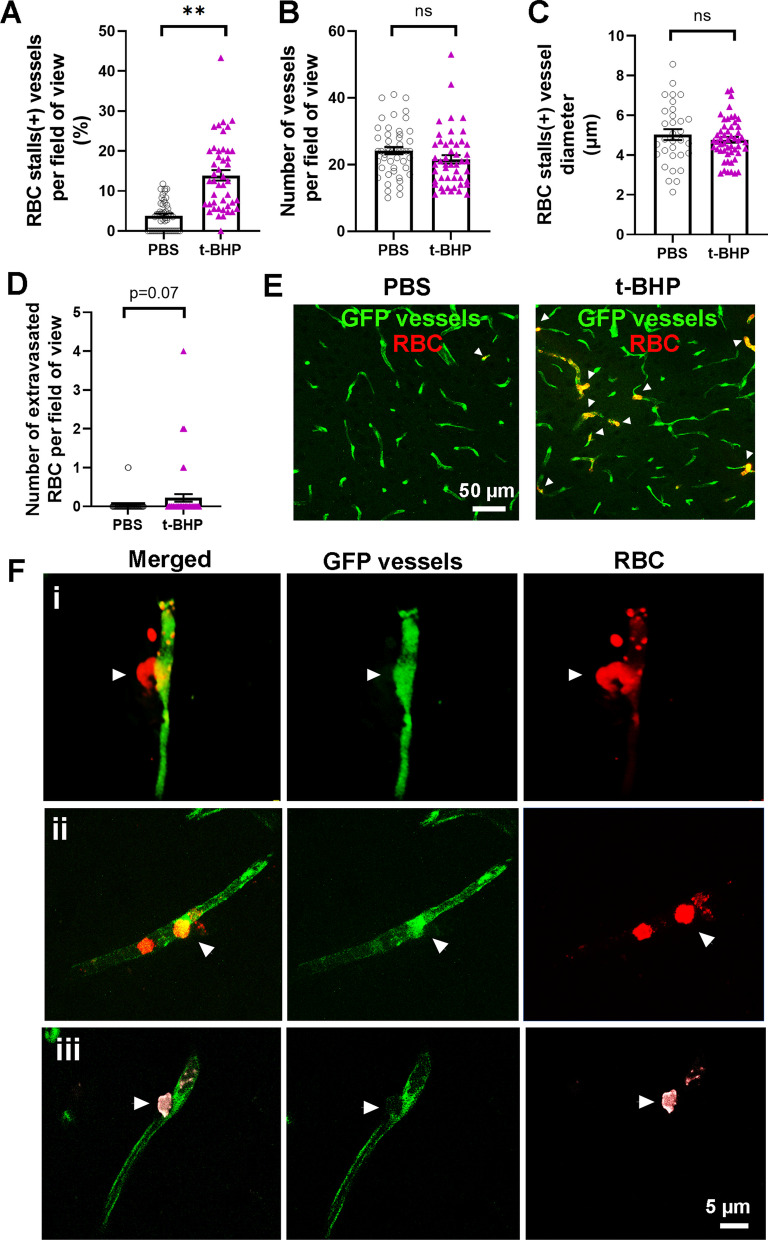
Post-mortem analysis of mouse brain sections shows increased t-BHP-RBC–brain endothelial interactions 24 h after RBC injection. NIH ImageJ quantification of the digitized images shows significantly higher percentage of blood vessels with RBC stalls in mice injected with t-BHP-treated RBC relative to control PBS-treated RBC (**A**). No difference is observed in the total number of blood vessels following control PBS- versus t-BHP-treated RBC injections (**B**), and the blood vessels with stalled RBC are capillaries (< 10 µm vessel diameter) (**C**). A trend of increased RBC extravasation is seen following the injection of t-BHP-treated RBC (**D**). Representative images showing RBC stalls in the t-BHP-RBC versus PBS-RBC injected mice (white arrowheads) (**E**). Scale bar = 50 µm in **E**. Representative images showing t-BHP-RBC extravasation from brain capillary endothelium into brain parenchyma **(F)**. The brain endothelium at the site of t-BHP-RBC extravasation appears intact in **Fi** and **Fii**, with some alterations in **Fiii**. Scale bar = 5 µm in **F**. Data are presented as mean ± SEM of individual images analyzed per experimental group. Statistical analysis was performed using the LME. **p < 0.01

Mouse brain sections were examined ex vivo following RBC injections to study the interactions between the PKH26-labeled RBC and the GFP-labeled brain endothelium. We first examined whether treatment of RBC with an oxidative stressor alters their interaction with brain endothelium compared to the control mice injected with PBS-treated RBC. 24 h after RBC injection, the brains of mice injected with t-BHP-treated RBC show significantly more cerebral vessels with RBC stalls compared to PBS-treated RBC-injected mice (3.8 ± 0.5% in the PBS-RBC mice vs. 13.9 ± 1.3% positive vessels in the t-BHP-RBC mice, *p* < 0.01) (Fig. 3A and E, Additional file 1: Figure S3). This increased vessel number with RBC stalls in mice injected with t-BHP-treated RBC is not accompanied by a change in the total number of cerebral blood vessels (vessels per field of view: 24.2 ± 1 in the PBS-RBC mice vs. 21.6 ± 1.2 in the t-BHP-RBC mice, Fig. 3B) or cerebral vessel diameter (5.0 ± 0.3 µm in the PBS-RBC mice vs. 4.8 ± 0.1 µm in the t-BHP-RBC mice, Fig. 3C). All cerebral vessels analyzed in the current study are brain capillaries with a diameter of < 10 µm. A trend (*p* = 0.07) of increased RBC extravasation into the brain parenchyma is observed in the t-BHP-treated RBC injected mice compared with the PBS-treated RBC injected mice (RBC per field of view: 0.03 ± 0.03 in the PBS-RBC mice vs. 0.2 ± 0.09 in the t-BHP-RBC mice, Fig. 3D). Representative images show significantly greater RBC stalls in the t-BHP-treated RBC injected mice relative to PBS-treated RBC injected mice (Fig. 3E) and t-BHP-RBC egress from the brain capillary endothelium (Fig. 3Fi-iii). Notably, the brain capillary endothelium appears intact during RBC egress in most cases (Fig. 3Fi-ii).

### Microglial reactions occur in close proximity to stalled RBC in t-BHP-treated RBC injected mice 24 h after RBC injection

**Fig. 4:**
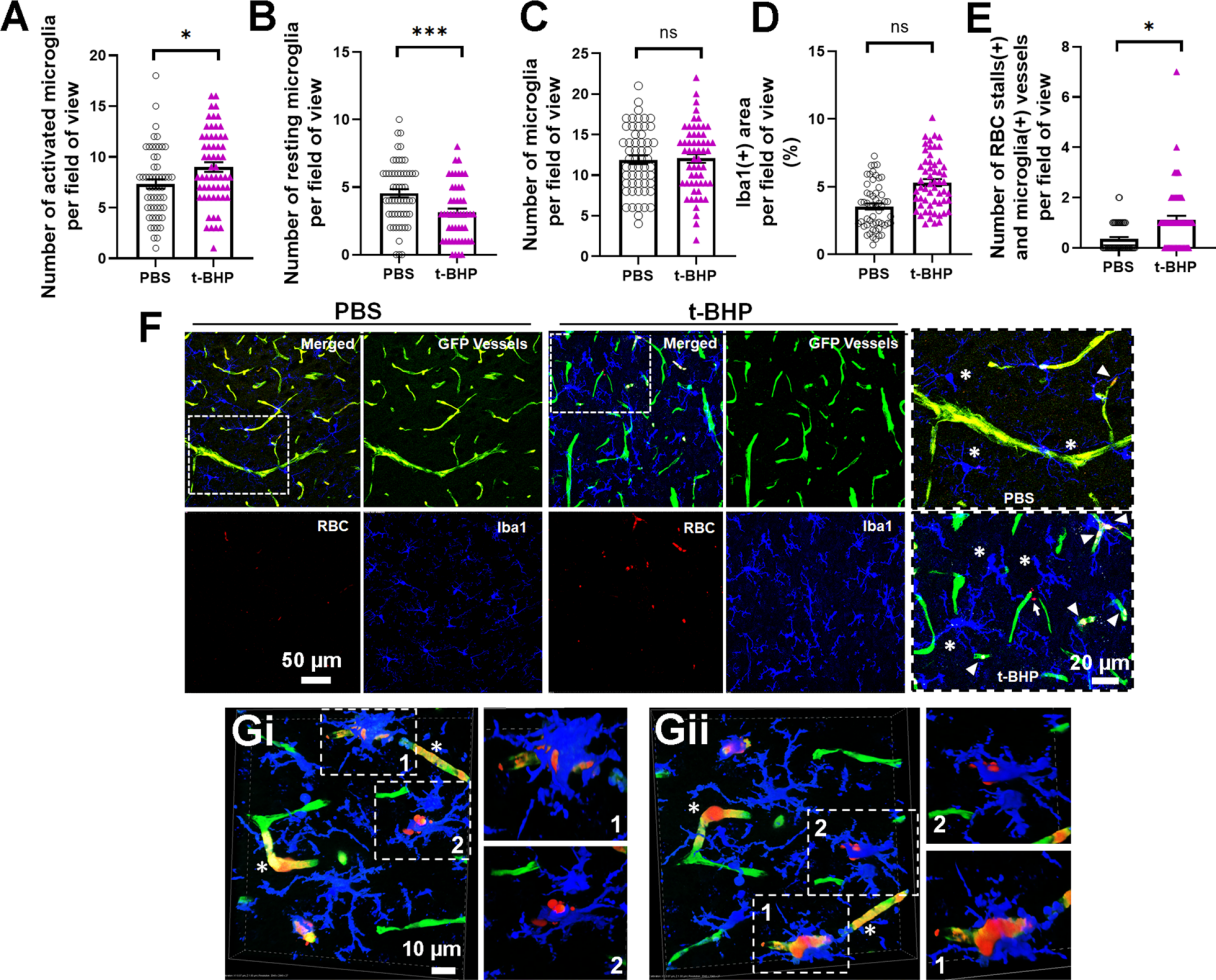
Microglial reactions occur in close proximity to cerebral vessels stalled with t-BHP-treated RBC 24 h after RBC injection. Following injection of t-BHP-treated RBC, the number of activated microglia in nearby brain tissue significantly increases (**A**) and the number of resting microglia significantly decreases (**B)**. The total number of microglia (**C**) and microglia-positive area (**D**) do not significantly differ between the control PBS-treated RBC and t-BHP-treated RBC-injected mice. Significantly more stalled RBC and microglia-positive vessels are measured in t-BHP-RBC-injected mice relative to control (**E**). Representative confocal images show GFP-positive blood vessels in green, PKH26-labeled RBC in red, and Iba1-positive microglia in blue (**F**), and show significantly greater RBC stalls and microglial activation in the t-BHP-RBC-injected mice. Scale bar = 50 or 20 µm in **F**. The dotted boxed areas in F are shown at higher resolution in the rightmost panel in F and show cerebral blood vessels with stalled RBC (white arrowheads) and extravasated RBC (white arrow). Microglia are labeled by asterisks (in **F**). Volume z-stack reconstructions of confocal images show RBC stalling and extravasation of t-BHP-treated RBC and microglial activation in brain tissue in close proximity to stalled RBC in cerebral blood vessel (**Gi** and **Gii** represent 180° rotation of the same image). The image shows stalled RBC in cerebral vessels (asterisks), activated microglia enveloping what appears to be a site of an extravasating t-BHP-treated RBC (**1**), and activated microglia enveloping a cluster of extravasated t-BHP-treated RBC (**2**). The dotted boxes (1 and 2) in Gi and Gii are shown at higher resolution for better visualization. Scale bar = 10 µm. Data are presented as mean ± SEM of individual images analyzed per experimental group. Statistical analysis was performed using the LME. *p < 0.05 and ***p < 0.001

The interplay between RBC, brain endothelium, and microglia was studied in mouse brain sections by performing immunostaining for Iba1, a protein marker of microglia and macrophages [28]. 24 h after RBC injection, the number of activated Iba1-positive microglia is significantly higher (microglia per field of view: 7.3 ± 0.5 in PBS-RBC mice vs. 9 ± 0.5 in the t-BHP-RBC mice, *p* < 0.05) (Fig. 4A, F, G) while the number of resting microglia is significantly lower (microglia per field of view: 4.5 ± 0.3 in PBS-RBC mice vs. 3 ± 0.3 in the t-BHP-RBC mice, *p* < 0.001) (Fig. 4B, F, G) in the mice injected with t-BHP-treated RBC relative to PBS-treated RBC. The total number of microglia (microglia per field of view: 11.9 ± 0.5 in PBS-RBC mice vs. 12 ± 0.5 in the t-BHP-RBC mice, Fig. 4C) and Iba1-positive immunoreactive area (% area: 3.5 ± 0.2 in PBS-RBC mice vs. 5.3 ± 0.3 in the t-BHP-RBC mice, Fig. 4D) are not statistically different between the PBS-RBC- and t-BHP-RBC injected mice. Further, the number of cerebral vessels that are positive for microglia and RBC stalls is significantly higher in the t-BHP-RBC-injected mice compared with PBS-RBC-injected mice 24 h post-injection (per field of view: 0.38 ± 0.07 in PBS-RBC mice vs. 1.1 ± 0.2 in the t-BHP-RBC mice, Fig. 4E). We observe significant microglial activation in the brains of the mice treated with t-BHP-RBC (Fig. 4G); in some instances, microglia envelope brain endothelium at sites that are positive for RBC stalls (Fig. 4G). Activated microglia are also seen enveloping a cluster of extravasated RBC in the brain parenchyma of t-BHP-RBC injected mice (Fig. 4G).

### Post-mortem assessment shows RBC–brain endothelial interactions and inflammation responses 7 days after t-BHP-treated RBC injection

**Fig. 5:**
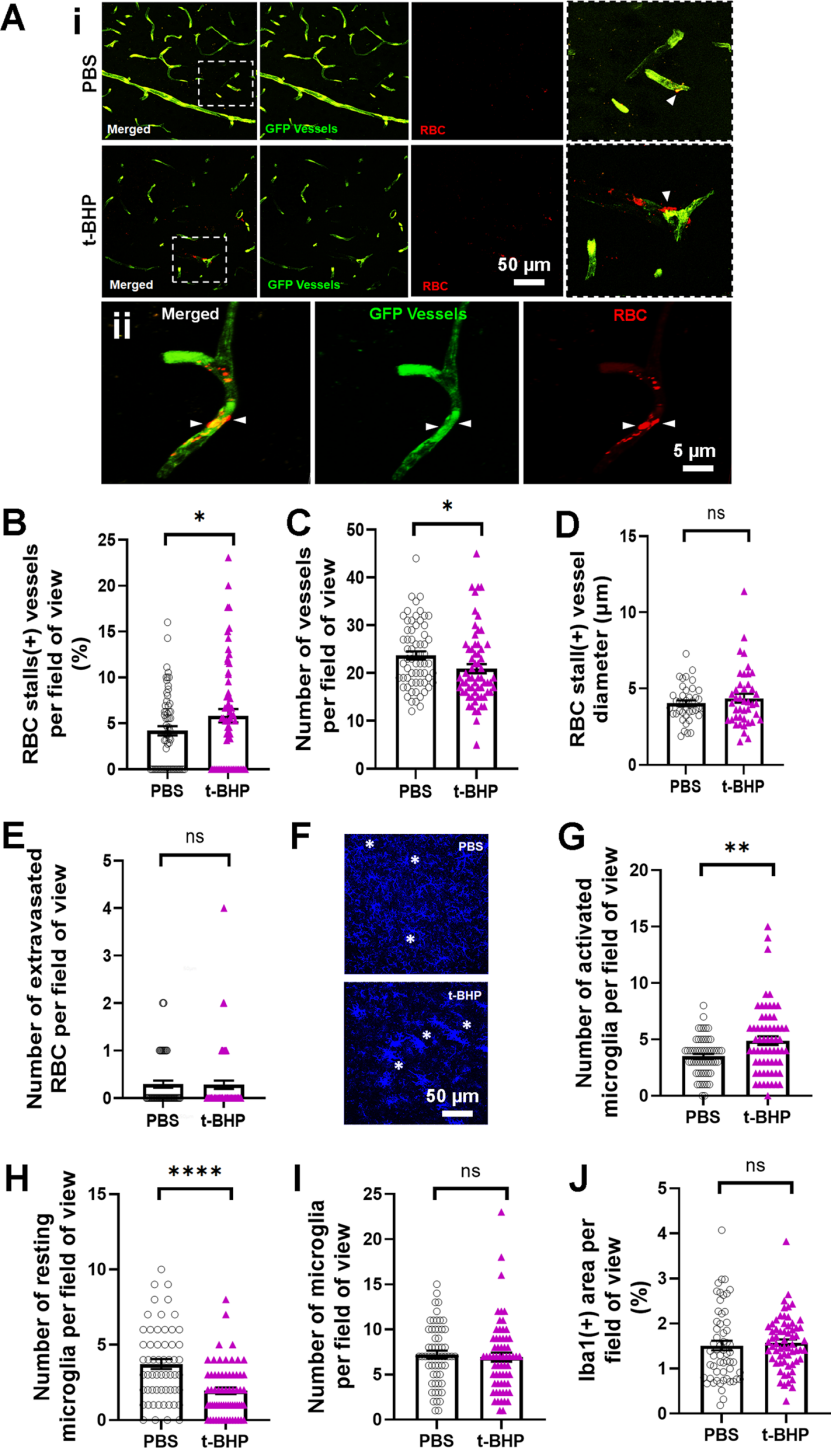
Post-mortem analysis of mouse brain section 7 days after RBC injections shows significantly more RBC stalls in cerebral blood vessels and higher brain inflammatory responses in the brain parenchyma following the injection of t-BHP-treated RBC. Representative confocal images show GFP-positive blood vessels in green and PKH26-labeled RBC in red (**Ai**). Scale bar = 50 µm in **Ai**. The dotted boxed areas in the merged channel in Ai are shown as higher resolution images in the rightmost panel in **Ai,** and cerebral blood vessels with stalled RBC are shown by white arrowheads **(Ai**). A confocal image showing t-BHP-RBC adhesion to and extravasation across brain capillary endothelium (**Aii**). The brain endothelium at the site of t-BHP-RBC extravasation appears intact in **Aii**. Scale bar = 5 µm in **Aii**. NIH ImageJ quantification of the digitized images shows significantly higher percentage of blood vessels with RBC stalls (**B**) and a lower number of total blood vessels in mice injected with t-BHP-treated RBC (**C**). The vessels showing stalled RBC are capillaries (< 10 µm blood vessel diameter) (**D**), and no significant difference is observed in the extravasated RBC between the groups under these assay conditions (**E**). Representative confocal images show Iba1-positive microglia (blue) in the PBS- and t-BHP-treated RBC-injected mice (**F**). Scale bar = 50 µm in **F**, and asterisks show resting microglia in the PBS-RBC-injected mice and activated microglia in the t-BHP-RBC-injected mice (**F**). Injection of t-BHP-treated RBC significantly increases numbers of Iba1-positive activated microglia (**G**) and significantly reduces numbers of Iba1-positive resting microglia (**H**), which indicate neuroinflammation in t-BHP-RBC-injected mice. The total number of microglia (**I**) and microglia-positive area (**J**) do not differ between the PBS- and t-BHP-RBC-injected groups. Data are presented as mean ± SEM of individual images analyzed per experimental group. Statistical analysis was performed using the LME. *p < 0.05, **p < 0.01, and ****p < 0.0001

To determine if the altered RBC–brain endothelial–microglial response observed 24 h after RBC injection is an acute or persistent response, we allowed the t-BHP-treated RBC to circulate for a week and performed post-mortem analysis 7 days after RBC injection. Similar as the results observed at 24 h, there are significantly more cerebral vessels with RBC stalls in the mice injected with t-BHP-treated RBC compared to PBS-RBC injected mice (vessels with RBC stalls: 4.1 ± 0.5% in PBS-RBC mice vs. 5.8 ± 0.7% in the t-BHP-RBC mice, *p* < 0.05) (Fig. 5A, B). The total number of cerebral vessels (vessels per field of view (23.7 ± 0.9 in PBS-RBC mice vs. 20.9 ± 0.9 in the t-BHP-RBC mice) is slightly but significantly lower in the mice injected with t-BHP-RBC (Fig. 5C), and the size of vessels with RBC stalls (4.0 ± 0.2 µm in PBS-RBC mice vs. 4.4 ± 0.3 µm in the t-BHP-RBC mice) does not differ between the PBS- and t-BHP-RBC-injected mice (Fig. 5D). Unlike at 24 h after injection, we do not observe similar trend of extravasated RBC in the brain parenchyma in mice injected with t-BHP-treated RBC relative to PBS-treated RBC mice (per field of view: 0.29 ± 0.07 in PBS-RBC mice vs. 0.28 ± 0.09 in the t-BHP-RBC mice) (Fig. 5E).

The inflammatory responses in the brain measured by microglia Iba1 staining persist at 7 days after the injection of t-BHP-treated RBC. The number of activated microglia continues to be significantly higher (microglia per field of view: 3.5 ± 0.2 in PBS-RBC mice vs. 4.9 ± 0.4 in the t-BHP-RBC mice, *p* < 0.01) (Fig. 5F, G), while the number of resting microglia continues to be significantly lower (microglia per field of view 3.7 ± 0.3 in PBS-RBC mice vs. 1.9 ± 0.2 in the t-BHP-RBC mice, *p* < 0.0001) (Fig. 5F and H) in the t-BHP-RBC mice compared with PBS-RBC mice. No changes in the total microglia number (microglia per field of view: 7.2 ± 0.4 in PBS-RBC mice vs. 6.9 ± 0.5 in the t-BHP-RBC mice, Fig. 5I) or Iba1-positive microglia immunoreactive area (% area: 1.5 ± 0.1 in PBS-RBC mice vs. 1.6 ± 0.07 in the t-BHP-RBC mice, Fig. 5J) are observed between the t-BHP-RBC mice and PBS-RBC mice. These results indicate that increased RBC–cerebral endothelium interaction and microglial responses persist at 7 days after t-BHP-RBC injection.

### Injection of t-BHP-treated RBC results in CMH development in the absence of BBB leakage

**Fig. 6:**
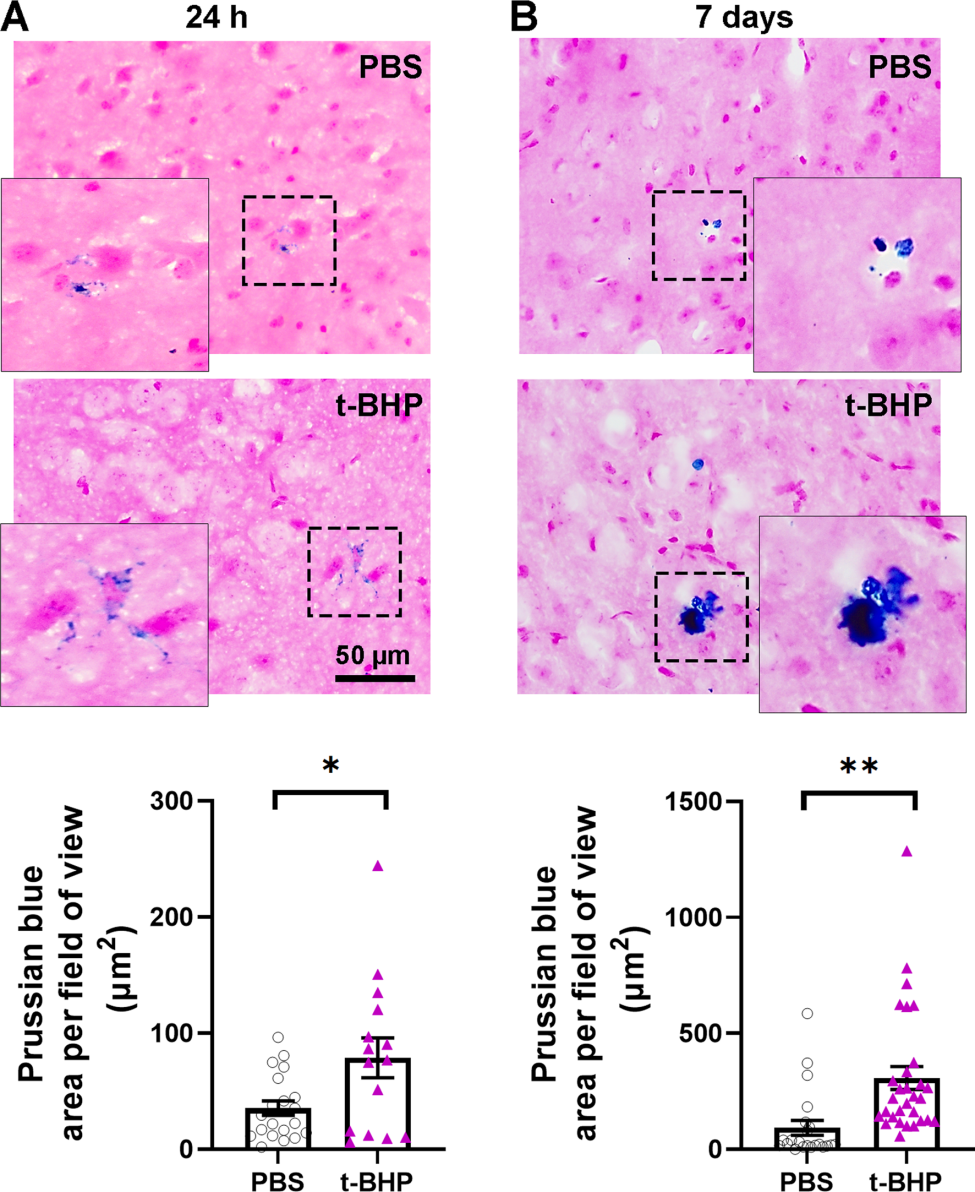
Significantly greater brain microhemorrhage load measured by Prussian blue in mice injected with t-BHP-treated RBC relative to controls at 24 h and 7 days after RBC injection. The Prussian blue-positive area is significantly higher in the mice injected with t-BHP-treated RBC both at 24 h (**A**) and 7 days (**B**) after RBC injection compared with the PBS-treated RBC injected mice. The dotted boxed area in A and B are shown in a higher resolution image directly left or right of the dotted box to highlight the Prussian blue-positive area. Note the difference in the Y-axis scale in **A** and **B**. Scale bar = 50 µm. Data are presented as mean ± SEM of individual images analyzed per experimental group. Statistical analysis was performed using the LME. *p < 0.05 and **p < 0.01

**Fig. 7:**
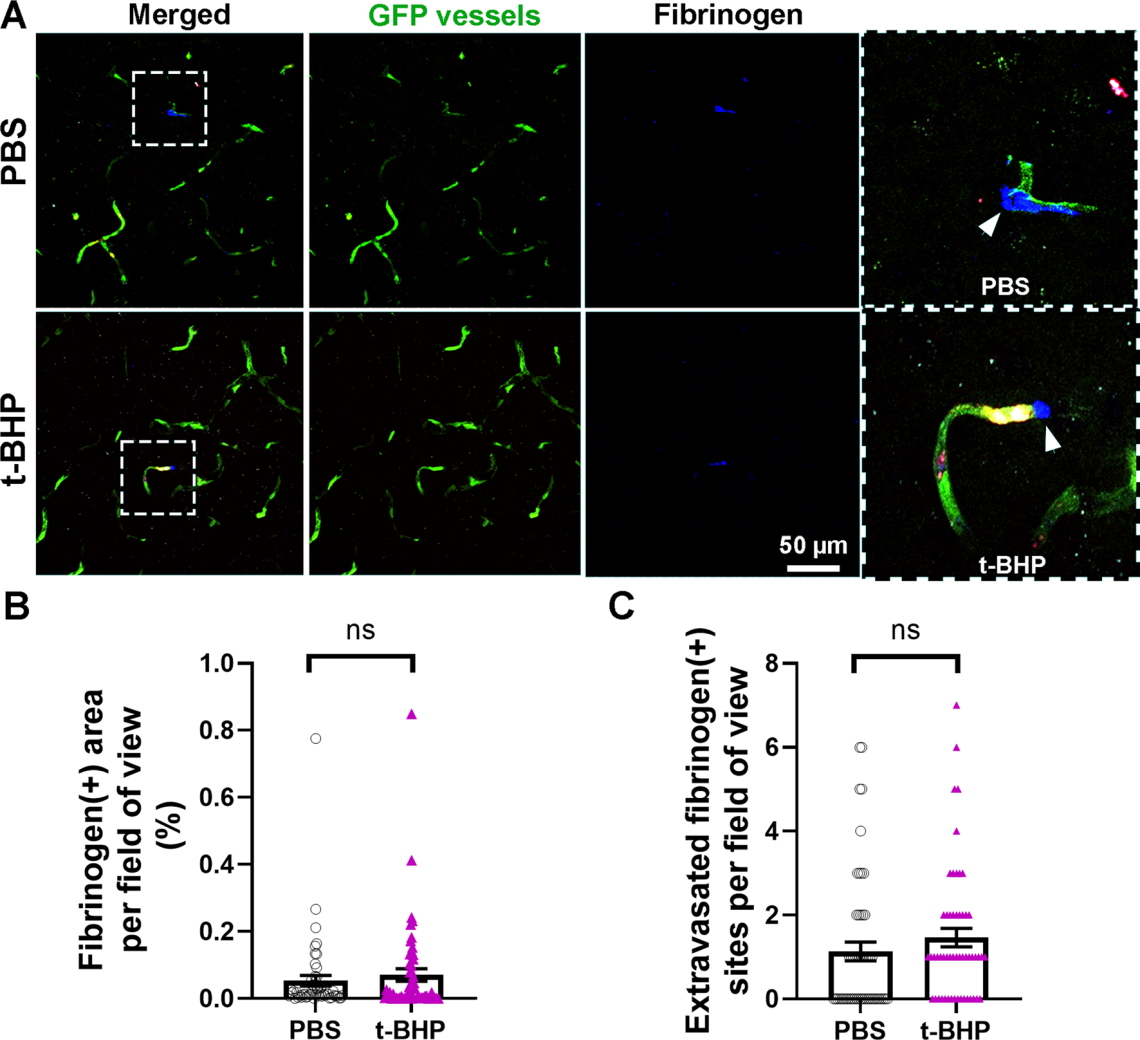
No significant difference in leaky cerebral blood vessels measured by fibrinogen immunostaining in post-mortem mouse brain section 24 h after injection of control versus t-BHP-treated RBC. Representative confocal images show GFP-positive blood vessels in green, PKH26-labeled RBC in red, and fibrinogen in blue (**A**). Scale bar = 50 µm. The dotted boxed areas in the merged channels in A are shown as higher resolution images in the rightmost panel in A for the PBS- and t-BHP-RBC-injected mice. White arrowheads show extravasated fibrinogen, a marker of BBB leakage (**A**). NIH ImageJ quantification of the digitized images shows no difference in the total fibrinogen-positive area (intra- and extra-vascular) (**B**) and extravasated fibrinogen-positive sites between the PBS- and t-BHP-RBC injected groups, which is a measure of BBB leakage (**C**). Data are presented as mean ± SEM of individual images analyzed per experimental group. Statistical analysis was performed using the LME

CMH in brain sections measured by Prussian blue staining for hemosiderin-iron show that t-BHP-RBC mice have significantly larger Prussian blue-positive area compared to PBS-RBC mice after both 24 h (35 ± 6 µm^2^ in PBS-RBC mice vs. 79 ± 17 µm^2^ in the t-BHP-RBC mice, *p* < 0.05, Fig. 6A and Additional file 1: Figure S4) and 7 days (93 ± 32 µm^2^ in PBS-RBC mice vs. 307 ± 50 µm^2^ in the t-BHP-RBC mice, *p* < 0.01, Fig. 6B and Additional file 1: Figure S4) of circulation of labeled RBC. Notably, the Prussian blue lesions are threefold larger at 7 days than at 24 h after RBC injection. Further, BBB leakage measured by fibrinogen immunostaining 24 h after RBC injection (Fig. 7) does not significantly differ between mice injected with t-BHP-treated RBC and controls injected with PBS-treated RBC. We measured both the intravascular and extravascular fibrinogen immunoreactivity combined (Fig. 7B), and the extravascular fibrinogen immunoreactivity alone (Fig. 7C); the latter is a measure of BBB leakage. No significant difference in the extravasated fibrinogen immunoreactivity in the brains of the mice injected with t-BHP-RBC is observed, suggesting that CMH develop following injection of t-BHP-treated RBC in the absence of BBB leakage.

## Discussion

Altered RBC–brain endothelial interactions and the ensuing thrombotic complications have been well-documented in infectious and vascular diseases, but the relevance of such altered interactions leading to RBC egress into the brain and subsequent cerebral microhemorrhaging has received relatively little attention. Our previous work shows robust adhesion and engulfment of t-BHP-treated RBC by the murine and human brain microvascular endothelial monolayers, which is associated with the migration of iron-rich RBC and/or their degradation product across intact brain endothelial monolayers in vitro [14, 15, 19]. These findings are relevant to CMH formation as iron-rich RBC or their degradation products can produce CMB signatures [11]. In the present study, using two-photon imaging, we demonstrate the temporal dynamics of t-BHP-treated RBC stalls in the cerebral blood vessels in vivo, and provide evidence of t-BHP-treated RBC extravasation into the brain parenchyma, subsequent microglial responses, and CMH formation in post-mortem mouse brain tissue. First, we show that t-BHP-treated RBC stall in the cerebral blood vessels very rapidly, as early as 1 h after RBC injection in mice, with most RBC stalls clearing by 24 h. Second, increased cerebral blood vessel stalls are accompanied by decreased blood flow velocity in mice injected with t-BHP-treated RBC, presumably due to decreased erythrocyte deformability after t-BHP stress [16]. Third, post-mortem analysis of brain sections corroborates these in vivo imaging findings and reveals that increased RBC–endothelium interactions are localized to the cerebral capillaries. Fourth, these altered RBC–brain endothelial interactions with t-BHP-treated RBC are associated with microglial activation and increased CMH burden, in the absence of BBB leaks, that persist up to 7 days after RBC injection. Overall, our data suggest that altered RBC–brain endothelial interactions can induce a persistent microglial response and CMH development.

RBC are the most abundant cells in the blood stream with a highly regulated life span. Within the blood circulation, RBC are constantly exposed to stresses and stimuli that trigger their removal from the blood circulation by a specialized process known as erythrophagocytosis. Aged and/or damaged RBC are recognized by professional phagocytes (macrophages) with subsequent erythrophagocytosis and clearance from the blood circulation [15, 29–31]. Besides professional phagocytes, there is increasing evidence that endothelial cells, including the cerebral endothelium, are also capable of phagocytosis [17, 32]. For example, in the case of embolic vessel occlusion, endothelial lamellipodia were found to surround emboli within hours, with subsequent engulfment of the emboli by brain endothelium followed by extravasation into the perivascular space. Extravasated emboli were eventually phagocytosed and degraded by brain parenchymal microglia [17]. Similarly, our previous in vitro work provides evidence for brain endothelial erythrophagocytosis and shows that oxidatively stressed RBC adhere to and are engulfed by murine and human brain endothelial cells [14, 15, 19]. Subsequent work has now shown altered brain–endothelial RBC interactions for *Plasmodium falciparum*-infected RBC in vivo [33] and glycated RBC in vitro [34]. Consistent with these observations, our in vivo two-photon microscopy work in the current study shows increased localization of t-BHP-treated RBC to the brain endothelium at 1–4 h after RBC injection which recovers over 7 days, compared with mice injected with PBS-RBC that show robust RBC flow within the cerebral vasculature. This is supported by optical coherence tomography imaging of cerebral capillary segments with stalling RBC by Erdener et al., who reported that RBC stalls are short-lived in healthy mice [35]. Stalls of the t-BHP-treated RBC in the cerebral vasculature are confirmed by closely examining the two-photon microscopy images, which reveal apparent colocalization of t-BHP-RBC (red) with the GFP-positive brain endothelial cells (green). Significant RBC stalls are observed in mice injected with t-BHP-treated RBC at 1–4 h after RBC injection. A few RBC stalls persist up to 24 h, while most of them have cleared within 24 h. By 7 days, no significant RBC stalls are observed by in vivo two-photon imaging. Post-mortem analysis of brain tissue sections supports the two-photon microscopy findings. We find significantly more cerebral blood vessels positive for RBC stalls in post-mortem analysis for mice injected with t-BHP-treated RBC relative to controls injected with PBS-RBC at both 24 h and 7 days after RBC injection; the small but significant increase at 7 days is not observed with two-photon microscopy, which may be due to the use of different imaging modalities (postmortem vs. intravital) and the measurements from different brain regions sampled (somatosensory cortex during the two-photon imaging and cortical and subcortical regions during post-mortem imaging). There appears to be a temporal decline in the RBC stalls as fewer t-BHP-treated stalled RBC are found in the cerebral blood vessels at 7 days, compared with 24 h after t-BHP-RBC injection. The exact mechanism of RBC clearance from the cerebral vasculature is unclear; while some RBC stalls may be dynamic and move along the vasculature with time, RBC that remain in contact with the endothelium may be cleared via brain endothelial erythrophagocytosis [14–17, 19], much like the process of angiophagy wherein larger fibrin clots and microspheres retained by brain microvessels, including arterioles and capillaries, are gradually cleared from the vasculature to recanalize blood vessels [17, 18, 32].

t-BHP-treated RBC, a well-studied model of RBC senescence [23], have reduced deformability [16], increased phosphatidylserine exposure [36], and reduced expression of CD47 [15]; these are key markers of RBC aging that are known to trigger erythrophagocytosis and RBC clearance. This enhanced clearance of t-BHP-treated RBC may account for the lower number of RBC in the blood circulation compared with PBS-treated RBC during the first 24 h. Apart from the significant stalls observed in the blood vessels of mice injected with t-BHP-treated RBC, we also find evidence for RBC attachment to, and extravasation across, the brain capillary endothelium. In some instances, clear egress of intact t-BHP-treated RBC from the brain capillaries is observed, with the capillary wall appearing intact at the site of RBC migration in most instances. Accordingly, fibrinogen immunostaining shows no significant difference in extravasated fibrinogen between the brain sections of mice injected with t-BHP-treated RBC and PBS-treated RBC, suggesting an absence of substantial BBB leakage. Quantification of RBC extravasation shows a strong trend toward greater extravasation of t-BHP-treated RBC from the brain capillaries 24 h after RBC injection. These findings are consistent with the migration of intact microspheres across the brain capillaries in rats [18]. Overall, the in vivo and post-mortem findings collectively show the temporal dynamics of RBC–brain endothelial interactions and subsequent mobilization of oxidatively stressed RBC across an intact brain capillary endothelium.

RBC stalling is associated with a significant reduction in the number of brain capillaries with t-BHP-treated RBC at 7 days. The exact mechanism involved in the reduction of brain capillaries is not clear. However, interaction of the t-BHP-treated RBC with the brain capillary and subsequent phagocytosis by the brain endothelial cells may increase intracellular iron load, which can trigger cellular apoptosis [15]. While our previous work and that of others show no change in BBB permeability with brain endothelial erythrophagocytosis [15, 34], a modest increase in markers of cellular apoptosis with endothelial erythrophagocytosis and increased BBB permeability with angiophagy have been reported by others [16, 18]. Another mechanism contributing to brain capillary reduction at 7 days after t-BHP-RBC injection may be elimination of brain capillaries that fail to regain blood flow due to persistent RBC stalling [37].

Though largely presumed to arise from extravasation of RBC due to disruption of cerebrovascular integrity, there is increasing evidence for CMH originating from other sources that increase brain iron—for example, alterations in brain iron homeostasis or non-hemorrhagic migration of iron-rich RBC into the perivascular space have been suggested to produce CMH [11, 13]. The altered RBC–brain endothelial interactions and subsequent migration of RBC or their iron-rich degradation products across the brain capillaries observed in our study are therefore relevant to the pathogenesis of CMH as discussed below. Abnormal RBC adhesion and engulfment by brain endothelium has been shown to be associated with increased intracellular and abluminal (brain-side) iron and iron-rich RBC degradation product hemoglobin [14, 15], which may produce CMH signatures. Alternatively, apparently intact RBC may be retained by and extravasate across brain endothelium. Once in the brain perivascular space, RBC can be degraded by microglial cells to release iron [38]. Consistent with these observations, we find a significantly increased Prussian blue-positive iron load in the brains of mice injected with t-BHP-treated RBC compared with mice injected with PBS-treated RBC, at both 24 h and 7 days after RBC injection. CMH load is threefold higher at 7 days than at 24 h after t-BHP-treated RBC injection, which may be due to temporal CMH expansion with continued altered RBC–brain capillary endothelial interactions and/or may reflect the delay in the release of iron following RBC degradation in brain parenchyma. Collectively, these results show that increased RBC–brain endothelial interactions are associated with CMH development in vivo. Notably, RBC alterations (including phosphatidylserine exposure and RBC membrane alterations, along with increased RBC adherence to the vascular endothelium, similar to those observed with t-BHP treatment) have been observed in chronic kidney disease and Alzheimer’s disease [39–42], pathological conditions with high CMB prevalence.

Microglia are resident brain immune cells that migrate towards the site of injury within the central nervous system. Once at the site of injury, microglia perform important functions including phagocytosis of invading pathogens and are also capable of sealing and repairing brain capillary lesions [43]. To elucidate the microglial response to altered RBC-endothelial interactions, Iba1 immunostaining was performed on mouse brain sections in the current study. We find a shift in the microglial morphology to a more amoeboid versus ramified appearance, suggestive of microglial activation [44], in the t-BHP-RBC-injected mice compared with the PBS-RBC-injected mice up to 7 days after RBC injection. Interestingly, activated microglia are seen enveloping a cluster of extravasated RBC in brain parenchyma and stalled cerebral capillaries in the t-BHP-RBC-injected mice. Further, cerebral capillaries with RBC stalls are frequently associated with juxtavascular microglial cells. These findings show that altered RBC–brain endothelial interactions appear to induce a robust microglial response and significant microglial association with the brain endothelium. Though such a microglial response following increased RBC–brain endothelial interactions has not been reported previously, these findings are consistent with studies showing altered microglial morphology and dynamics with changes in cerebral blood flow [45] and with angiophagy [18]. A similar observation has been reported in a laser-induced CMH mouse model wherein microglia are found to envelop and repair microbleeding vessels, and stimulating microglial response to CMH attenuates vessel leakage, suggesting a protective role of microglia in CMH development [46]. Further, in a model of hemorrhagic arteriolar CMH, microhemorrhaging is associated with a rapid inflammatory response involving the migration of microglia toward the CMH location in vivo [47]. However, apart from the protective effects of microglia in CMH development, our previous work shows that microglia are associated with increased CMH burden [1, 48, 49]. Furthermore, microglial depletion was found to reduce microhemorrhaging in a mouse model of chronic kidney disease [1]. Therefore, microglia appear to serve dual roles in the context of CMH development, with some evidence suggesting a protective role [46] while other evidence indicates a shift towards a role of inflammation instead of protection [1, 48, 49].

The results from the current study raise some intriguing questions that need further investigation. First, it is critical to identify how cerebral endothelium engulf RBC and how RBC extravasate in vivo. Since RBC extravasation is a dynamic process and our post-mortem data represent only a snapshot at 24 h, evaluation of the brain tissue as well as in vivo imaging at earlier time points may be needed. Second, the enhanced interactions of oxidatively stressed RBC to cerebral blood vessels are associated with significantly decreased cerebral blood flow velocity early after RBC injection that normalize by 5 days after RBC injection. Note that the blood flow velocity measurements only reflect the movement of RBC in brain capillaries, and do not measure changes in cerebral blood flow (tissue perfusion) resulting from a reduction in the number of brain capillaries. In some instances, we observe dramatic RBC stalls obstructing a capillary network in mice injected with t-BHP-treated RBC. Could these RBC stalls lead to hypoperfusion and subsequent localized ischemia, and does brain endothelial erythrophagocytosis represent an RBC-stalled vessel clearance mechanism? A recent study shows that RBC aggregation at the cerebral artery level leads to thrombosis-induced ischemia and CMH development [50]. To rule out thrombus formation, we used intravascular fibrin/fibrinogen immunostaining and do not find increased vascular fibrin/fibrinogen immunoreactivity in the mice injected with t-BHP-treated RBC in the current study. Third, we used post-mortem brain sections to assess BBB injury using fibrinogen immunostaining. Due to the cross-sectional nature of the analysis, we cannot rule out transient BBB changes during the course of the study. Moreover, fibrinogen has a large molecular weight (> 340 kDa), making it an appropriate marker to detect large BBB permeability changes that might be involved in RBC passage. It is possible that smaller BBB leaks were not detected in the present study. Fourth, RBC in the current study were chemically aged to mimic some of the key changes observed with RBC aging, including reduced deformability, phosphatidylserine exposure, and reduced CD47 expression. Future work will need to focus on naturally aged RBC isolated by methods such as density centrifugation or utilize RBC from mouse models with known RBC alterations such as sickle cell mice [51] or chronic kidney disease [39, 41]. Finally, Iba1 is also a marker of macrophages [52], and the involvement of microglia versus macrophages (brain resident or systemic) [53] was not differentiated in the current study.

## Conclusions

**Fig. 8:**
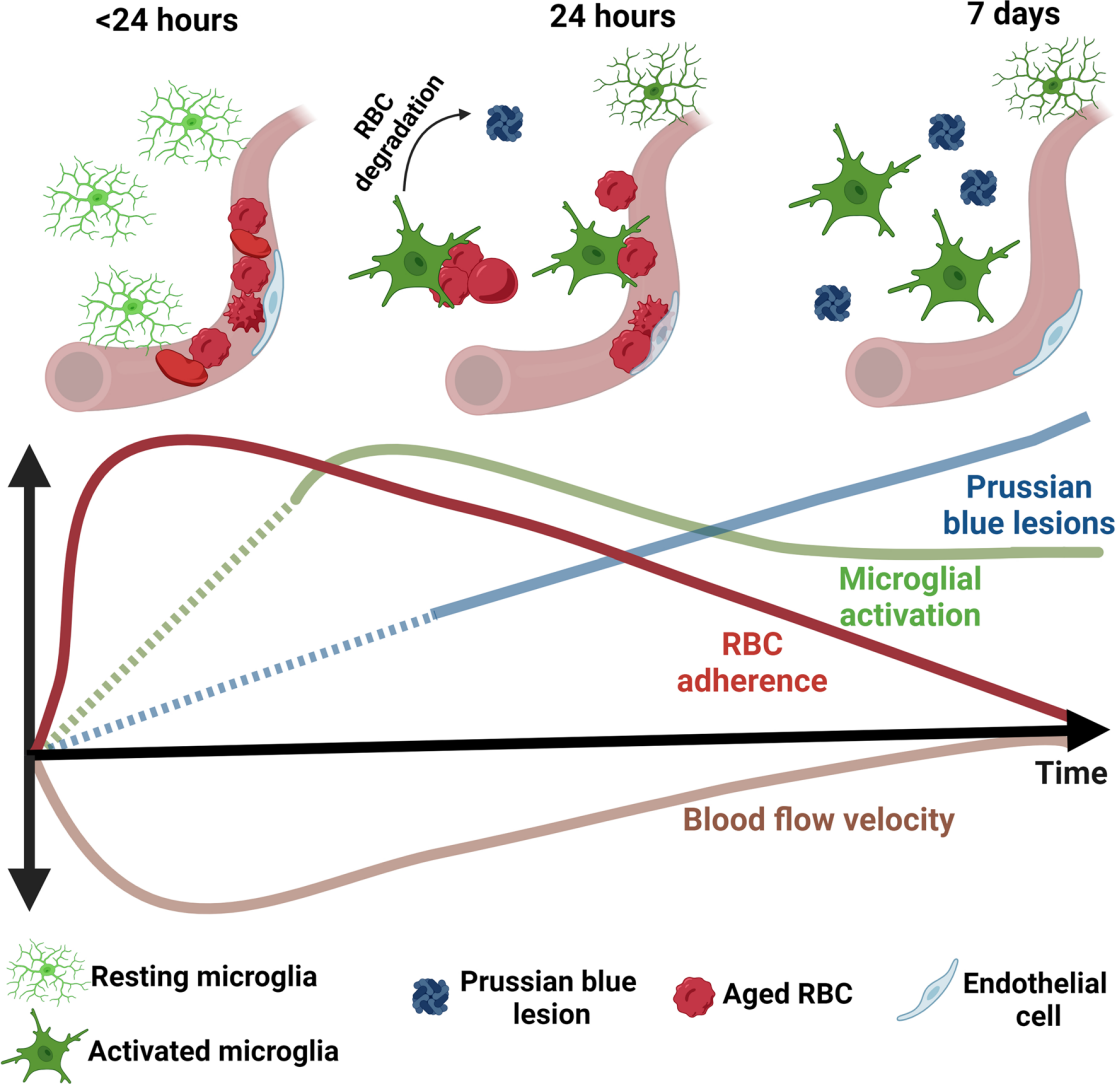
Schematic of the temporal dynamics of t-BHP-treated RBC interactions with cerebral vasculature and subsequent microglial responses and cerebral microhemorrhage. t-BHP-treated RBC stall in the cerebral capillaries early after RBC injection, and are eventually cleared. Stalled RBC in cerebral capillaries coincide with decreased cerebral blood flow velocity, which recovers over time as RBC adhere to and extravasate from the brain capillary endothelium into the brain perivascular space. Increased RBC–brain capillary interactions/RBC stalling triggers microglial activation and cerebral microhemorrhage. The dotted lines indicate predicted profiles and were not studied in the current study

Our results show that RBC damaged by oxidative stress associate with brain capillary endothelium, and these altered RBC–brain capillary endothelial interactions (RBC stalls) are dynamic and resolve over time as RBC extravasate into brain parenchyma (Fig. 8). These altered RBC–brain capillary interactions coincide with reduced cerebral blood flow velocity, persistent microglial activation, and increased CMH load in the absence of overt BBB leakage. Increased RBC interactions with brain capillary endothelium may therefore represent an alternate source of CMH development distinct from the traditional view of RBC extravasation in brain parenchyma due to microvascular disruption, and may be clinically relevant for conditions associated with increased RBC stress such as sickle cell disease [51], chronic kidney disease [39, 41] and perhaps aged RBC.

### Supplementary Information

**Additional file 1: Figure S1. **Images of RBC stalls in mice injected with t-BHP-treated but not PBS-treated RBC. **Figure S2.** Additional examples of t-BHP-treated RBC stalls in vessels at 4 h and the clearance of the stalled RBC at 24 h after injection. **Figure S3.** Example of dramatic RBC stalls in cerebral capillaries in the brain sections of t-BHP-treated RBC-injected mice. **Figure S4.** Additional examples of Prussian blue-positive stains in mice injected with PBS- and t-BHP-treated RBC at 24 h and 7 days after RBC injection.

**Additional file 2: Video 1.** PBS-treated RBC flow through a capillary at 1 h after RBC injection. Note that control RBC can stall in the blood vessel temporarily.

**Additional file 3: Video 2.** PBS-treated RBC flow through the same capillary shown in Video 1 at 24 h after RBC injection.

**Additional file 4: Video 3.** t-BHP-treated RBC stall in capillaries at 4 h after RBC injection.

**Additional file 5: Video 4.** t-BHP-treated RBC stall in the same capillaries as shown in Video 3 at 24 h after RBC injection.

**Additional file 6: Video 5.** t-BHP-treated RBC stalls in a capillary at 4 h after RBC injection, and some RBC flow slowly through the capillary.

**Additional file 7: Video 6.** The RBC stall shown in Video 5 is cleared at 24 h.

**Additional file 8: Video 7.** t-BHP-treated RBC stall in capillaries at 4 h after RBC injection.

**Additional file 9: Video 8.** The RBC stall in one capillary (shown in Video 7) is cleared while the other persists at 24 h after RBC injection.

**Additional file 10: Video 9.** t-BHP-treated RBC stalled in a large blood vessel wall and also in nearby capillaries.

## Data Availability

Data and materials are available from the corresponding authors on reasonable request.

## Abbreviations

BBB: Blood–brain barrier
CMB: Cerebral microbleed(s)
CMH: Cerebral microhemorrhage(s)
GFP: Green fluorescent protein
Iba1: Ionized calcium-binding adapter molecule1
LME: Linear mixed effects
MRI: Magnetic resonance imaging
PBS: Phosphate buffered saline
RBC: Red blood cell(s)
t-BHP: Tert-butylhydroperoxide
