# Supplementary material for: Erythrocyte–brain endothelial interactions induce microglial responses and cerebral microhemorrhages in vivo
Source: J Neuroinflammation. 2023 Nov 15;20:265. doi: 10.1186/s12974-023-02932-5 (PMC10647121; doi:10.1186/s12974-023-02932-5)
Supplement: Supplementary file 1 — Additional file 1: Figure S1. Images of RBC stalls in mice injected with t-BHP-treated but not PBS-treated RBC. Figure S2. Additional examples of t-BHP-treated RBC stalls in vessels at 4 h and the clearance of the stalled RBC at 24 h after injection. Figure S3. Example of dramatic RBC stalls in cerebral capillaries in the brain sections of t-BHP-treated RBC-injected mice. Figure S4. Additional examples of Prussian blue-positive stains in mice injected with PBS- and t-BHP-treated RBC at 24 h and 7 days after RBC injection. [file 12974_2023_2932_MOESM1_ESM.docx]

**Additional Figures**


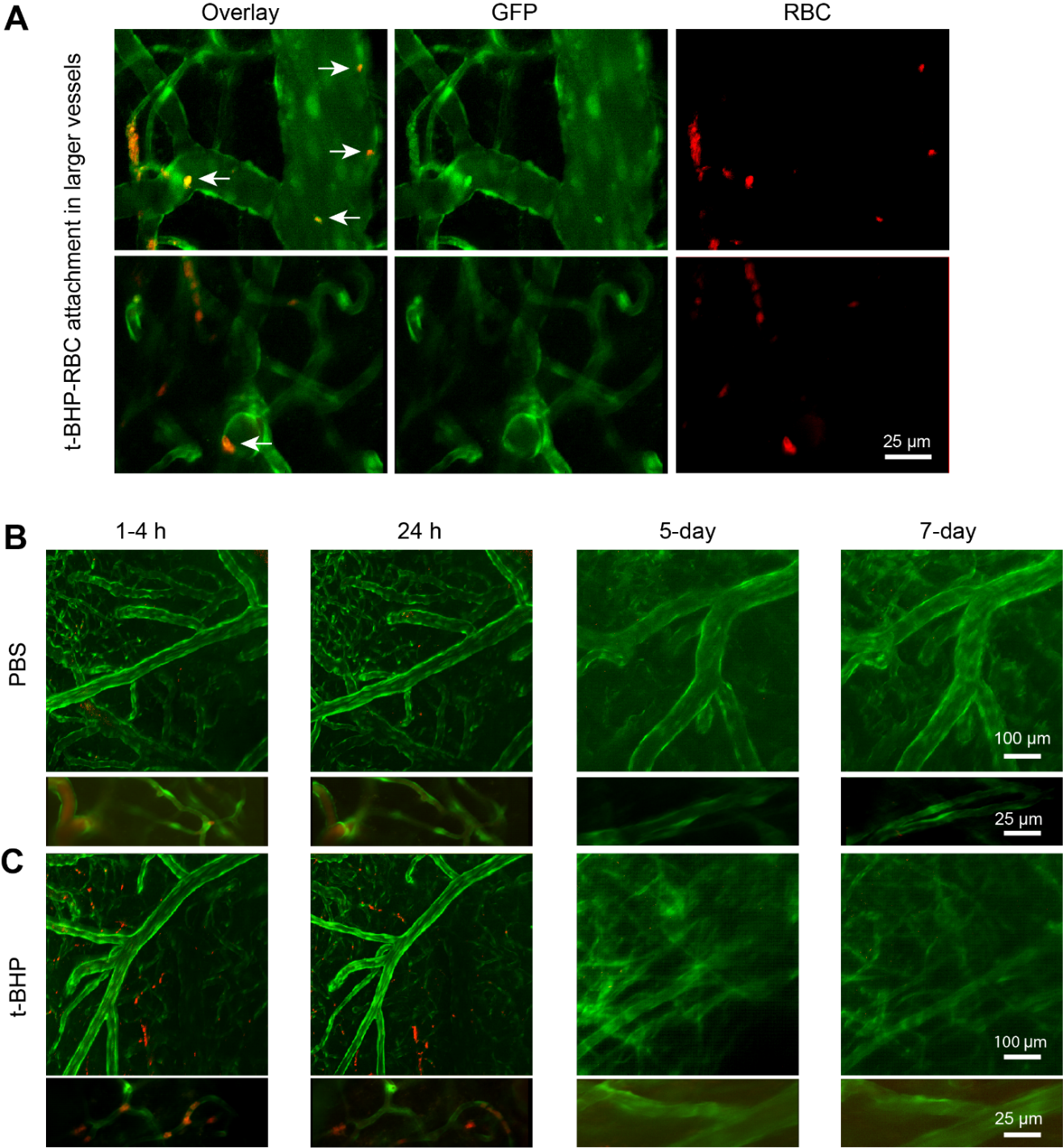


**Additional Figure S1**. **Images of RBC stalls in mice injected with t-BHP-treated but not PBS-treated RBC**. t-BHP-treated RBC (red) attach in larger blood vessels (green) shown by arrows (**A**). Representative *in vivo* two-photon images of blood vessels (in green) and RBC stalls (in red) at 1-4 h, 24 h, 5-days and 7-days after injection of PBS-treated RBC (**B**) or t-BHP-treated RBC (**C**) in Tie-2 GFP mice.


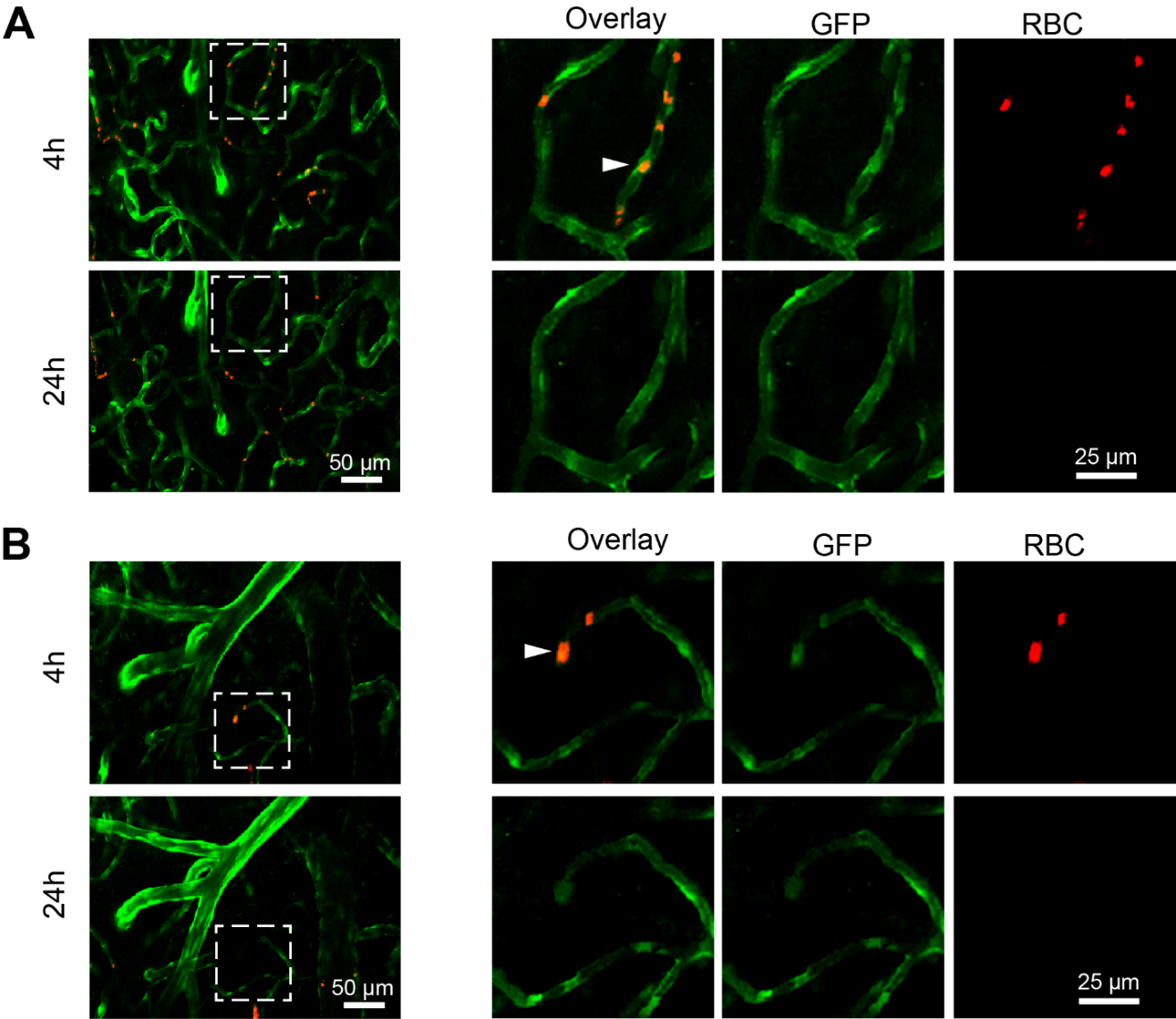


**Additional Figure S2. Additional examples of t-BHP-treated RBC stalls in vessels at 4 h and the clearance of the stalled RBC at 24 h after injection** (**A, B**, arrowheads).


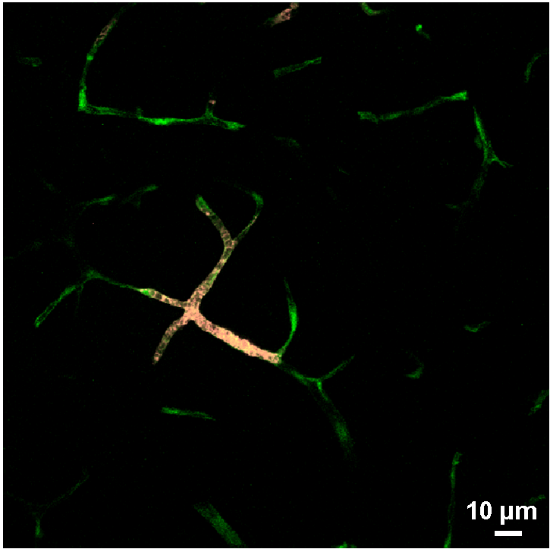


**Additional Figure S3: Example of dramatic RBC stalls in cerebral capillaries in the brain sections of t-BHP-RBC injected mice**.


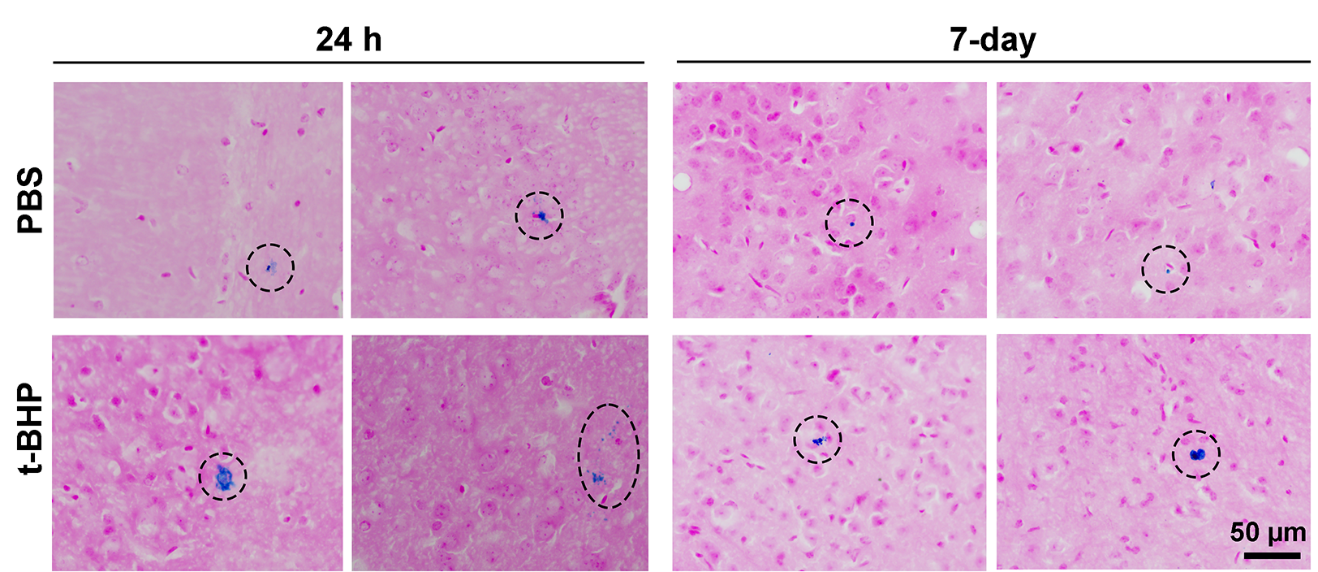


**Additional Figure S4: Additional examples of Prussian blue-positive stains in mice injected with PBS- and t-BHP-treated RBC at 24 h and 7 days after RBC injection**.

**Additional videos**

Video 1. PBS-treated RBC flow through a capillary at 1 h after RBC injection. Note that control RBC can stall in the blood vessel temporarily.

Video 2. PBS-treated RBC flow through the same capillary shown in Video 2 at 24 h after RBC injection.

Video 3. t-BHP-treated RBC stall in capillaries at 4 h after RBC injection.

Video 4. t-BHP-treated RBC stall in the same capillaries as shown in Video 3 at 24 h after RBC injection.

Video 5. t-BHP-treated RBC stalls in a capillary at 4 h after RBC injection, and some RBC flow slowly through the capillary.

Video 6. The RBC stall shown in Video 5 is cleared at 24 h.

Video 7. t-BHP-treated RBC stall in capillaries at 4 h after RBC injection.

Video 8. The RBC stall in one capillary (shown in Video 7) is cleared while the other persists at 24 h after RBC injection.

Video 9. t-BHP-treated RBC stalled in a large blood vessel wall and also in nearby capillaries.
